# The influence of salinity on the effects of Multi-walled carbon nanotubes on polychaetes

**DOI:** 10.1038/s41598-018-26729-2

**Published:** 2018-06-05

**Authors:** Lucia De Marchi, Victor Neto, Carlo Pretti, Etelvina Figueira, Federica Chiellini, Andrea Morelli, Amadeu M. V. M. Soares, Rosa Freitas

**Affiliations:** 1 Department of Biology & Center for Environmental and Marine Studies (CESAM), University of Aveiro, 3810-193 Aveiro, Portugal; 2 Department of Mechanical Engineering & Center for Mechanical Technology and Automation (TEMA), University of Aveiro, 3810-193 Aveiro, Portugal; 3 Department of Veterinary Sciences, University of Pisa, San Piero a Grado, Pisa, 56122 Italy; 4 Department of Chemistry and Industrial Chemistry, University of Pisa, Udr INSTM Pisa, Pisa, 56126 Italy

## Abstract

Salinity shifts in estuarine and coastal areas are becoming a topic of concern and are one of the main factors influencing nanoparticles behaviour in the environment. For this reason, the impacts of multi-walled carbon nanotubes (MWCNTs) under different seawater salinity conditions were evaluated on the common ragworm *Hediste diversicolor*, a polychaete species widely used as bioindicator of estuarine environmental quality. An innovative method to assess the presence of MWCNT aggregates in the sediments was used for the first time. Biomarkers approach was used to evaluate the metabolic capacity, oxidative status and neurotoxicity of polychaetes after long-term exposure. The results revealed an alteration of energy-related responses in contaminated polychaetes under both salinity conditions, resulting in an increase of metabolism and expenditure of their energy reserves (lower glycogen and protein contents). Moreover, a concentration-dependent toxicity (higher lipid peroxidation, lower ratio between reduced and oxidized glutathione and activation of antioxidant defences and biotransformation mechanisms) was observed in *H*. *diversicolor*, especially when exposed to low salinity. Additionally, neurotoxicity was observed by inhibition of Cholinesterases activity in organisms exposed to MWCNTs at both salinities.

## Introduction

Estuaries and shallow water bodies are among the most vulnerable aquatic ecosystems due to continuous influx of different contaminants commonly associated with agricultural and/or industrial activities^1–6^. These human activities can cause high environmental threats as these areas are key habitats for many ecologically relevant species including polychaetes^7^. The common ragworm *Hediste diversicolor* (O.F. Müller, 1776) is one of the most important and abundant species inhabiting intertidal mudflats of estuaries and shallow water bodies of the Atlantic and Indian Oceans^8^ as well as the Mediterranean Sea^9^, where they play an important ecological role^10,11^ and represent an important economic resource^12,13^. Moreover, these omnivorous sediment dwelling organisms are considered key species due to the remobilization of contaminants and nutrients associated to their burrowing and dietary behaviour^14–16^. This confers to *H*. *diversicolor* the capacity to act as bioindicator species of estuarine environmental quality. In particular, this species has been commonly used to assess pollution impacts due to metals^17–20^, polycyclic aromatic hydrocarbons^10,21^, pharmaceuticals^22,23^ and more recently engineered nanoparticles (ENMs)^11,24–30^. Nevertheless, studies assessing the toxic effects of carbon nanomaterials (CNMs) induced on this species and polychaetes in general, are limited^31,32^.

Currently, carbon nanotubes (CNTs) are one of the most important CNMs^33^. CNTs are hollow graphene cylinders that are microns to millimetres in length and they can be divided in single-walled (SWCNTs) with a diameter of 0.7 to 3 nm, and multi-walled (MWCNTs) with a diameter of 10 to 25 nm^34^. Due to their low mass density, high mechanical strength, high electron/hole mobility, and high thermal conductivity^34^, the industrial production and applications of CNTs are increasing rapidly^35^ and environmental exposure is therefore likely expected to increase. Releasing CNTs in the environment inevitably will cause their entrance into the aquatic systems through general weathering, disposals containing consumer products, accidental spillages, and waste discharges^36^. Due to their hydrophobic and non-biodegradable characteristics^37^, they can be accumulated by aquatic biota through body surface, digestive and respiratory systems^38^. The toxicity of CNTs or CNMs in general, does not only depend on their physico-chemical properties, but it is important to take in consideration different media in which CNTs are dispersed^39^. In particular, changes in the salinity of the aqueous environment can influence the NMs’ stability, altering their toxicity towards organisms^39^. As already demostrated^40,41^, the high surface area to volume ratio of NMs results in high surface reactivity, leads to structure changes in salt aqueous solutions, inducing modifications of biological membranes such as the expression of different cellular macromolecules (lipids or proteins)^42^.

Salinity also plays a fundamental role in estuarine systems. Specifically these enviroments can be dominated by episodic, short-lived freshwater inflows during storms that flush salt water out of the estuarine system^43^. These changes can lead to different areas of the estuary transiting from a highly stratified salt wedge, a partially mixed estuary, or possibly to a vertically homogeneous estuary, impacting inhabiting organisms, which are highly sensitive to salinity thresholds and will respond to altered salinity gradients^43^. In a climate change scenario, precipitation patterns and freshwater runoff, sea surface temperatures, evaporation, wind and solar radiation and other additional forcing factors influence the biological nature of estuarine landforms, habitats and ecosystems^43^. Particularly, hyposaline stress is predicted to become an urgent issue for the future^44^. In fact, extreme weather events may strongly decrease environmental salinity conditions, particularly in estuarine and coastal areas, where changes in seawater salinity are of major concern^45–47^.

Although polychaetes are able to tolerate great variations of salinity with the capacity to settle in naturally fluctuating environments such as estuaries^48,49^, few information is available on the combined effects of salinity and CNTs  in *H*. *diversicolor*, namely on organisms’ biochemical performance. Thus, in the present study biochemical responses (oxidative stress, metabolic, energetic and neurotoxicity related markers) resulting from impacts of two different MWCNT concentrations (0.10 and 1.00 mg/L), under two salinity levels (28-control, 21) were evaluated in *H*. *diversicolor* polychaetes after an exposure period of 28 days. The range of salinity used was selected: (i) according to the environmental salinity range where specimens were collected^50,51^ and (ii) the fact that salinity 21 did not have a negative impact on physiological and biochemical responses in *H*. *diversicolor*^48,49^, which allowed to evaluate the hypothesis that MWCNTs toxicity could depend only on the bioavailability of the CNMs and not on polychates sensitivity to low salinity conditions.

Regarding MWCNTs, the exposure concentrations were selected (i) considering the Predicted Environmental Concentrations (PECs) of CNTs in aqueous systems reported from the most recent literature^52^ which are projected to approximately 0.001–1000 µg/L^53^ and (ii) considering previous studies conducted using similar range of CNMs concentrations on vertebrate^54^ and invertebrate^32,55,56^ species.

## Materials and Methods

### Sampling and experimental conditions

*H*. *diversicolor* specimens were collected from the Mira channel, a low contaminated area at the Ria de Aveiro lagoon (Portugal)^57,58^. Organisms with similar weight (0.49 ± 0.2 g) were used to prevent differences on biochemical responses.

In the laboratory, specimens were placed in 5 different aquaria (20 L each) for 2 weeks. Aquaria were filled with a mixture of fine and medium sediment from the sampling area (a clean sampling area with sediment median value 1.59; percentage of fines 6.75 ± 0.79; percentage of organic matter content 3.24 ± 0.44), approximately 1/3 of the height of the aquarium, and artificial seawater (salinity 28) set up by the addition of artificial sea salt (Tropic Marin® Sea Salt) to deionized water, one day prior to utilization. During the acclimation period, organisms were maintained under constant photoperiod of 12 h light: 12 h dark, temperature (18 ± 1 °C), pH 7.9 and constant aeration. To respect species feeding requirements, during this period every two-three days specimens were fed *ad libitum* with commercial fish food (48.6% protein and 7.7% fat)^59^.

After the acclimation period, organisms were exposed for 28 days to two different salinities (21 and 28-control), each one combined with two different MWCNT concentrations (0.10 and 1.00 mg/L). For each condition, 3 aquaria were used with 10 organisms *per* aquarium. Each aquarium was set up at the same pH, temperature and aeration conditions as in the acclimation period (see above).

Prior to experiment initiation, the salinity was progressively decreased (1–2 units) every 2 days until testing value was reached (salinity 21). Both MWCNT concentrations and salinity levels were re-established weekly after complete water renewals to ensure the same exposure concentrations during the experiment. To promote stable suspension in the water column^60^, the MWCNTs were sonicated for 1 h using an ultrasound probe at 30 Hz (IKA Labortechnik IKASONIC U50). The added CNTs were homogenously dispersed in the water using one submersible circulation pump *per* aquarium, which diminishes the possibility that the dynamical equilibrium between gravitational settling and Brownian motion can result in the presence of CNTs near the sediment–water interface^61^.

### MWCNTs characterization

#### MWCNTS in water

**Figure 1:**
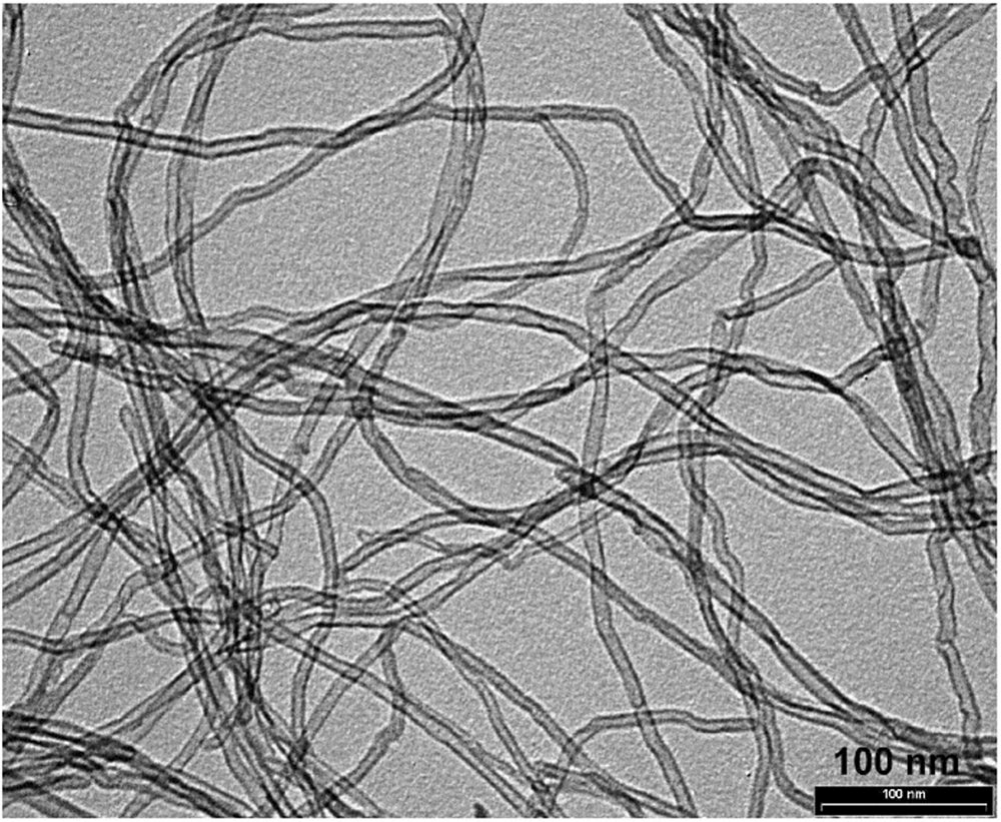
Transmission electromicrographs (TEM) of the powder form of MWCNTs produced via the catalytic carbon vapor deposition (CCVD) process.

Thin MWCNT materials were produced via the Catalytic Chemical Vapor Deposition (CCVD) process and characterized using Transmission electromicrographs (TEM) (Fig. 1). These CNMs were purchased from Nanocyl S.A. (MWCNTs: NC7000 series, http://www.nanocyl.com) and manufacturer’s specifications are showed in Table 1.

**Table 1:** Characterization of the powder form of MWCNTs.

Property	Unit	Value	Method of Measurement
Average Diameter	nanometers	9.5	TEM
Average Length	microns	1.5	TEM
Carbon Purity	%	90	TGA
Metal Oxide	%	10	TGA
Amorphous Carbon	—	*	HRTEM
Surface Area	m^2^/g	250–300	BET

*Pyrolytically deposited carbon on the surface of MWCNTs.

The concentrations of MWCNT used in this study were 0.10 and 1.00 mg/L prepared from a stock solution of 50 mg/L concentration. Although the Environmentally Relevant Concentrations (ERCs) of CNTs in water (based on a stochastic/probabilistic material-flow computer model) are in the range of µg/L or ng/L ^62^, the concentrations tested were chosen considering  the PECs of CNTs in aqueous systems reported from the most recent literature^52,53^, projected to approximately 0.001–1000 µg/L. These concentrations have been shown to produce cytotoxic, genotoxic, inflammatory and oxidative stress responses in different organisms^63,64^ as a consequence of the presence of amorphous carbon fragments which can induce higher levels of toxicity to biological systems in comparison to other metal NMs^65^.

The average size distribution and the polydispersity index (PDI) of MWCNT suspensions were analysed in each CNMs exposure condition (control-28 + 0.10 mg/L MWCNTs; control-28 + 1.00 mg/L MWCNTs; 21 + 0.10 mg/L MWCNTs; 21 + 1.00 mg/L MWCNTs) and at different exposure periods: t0: time zero, immediately after medium contamination (dispersed MWCNT concentrations in artificial seawater); t7: water samples collected after 1 week of exposure, before water renewal; t28: water samples collected after fourth week of exposure, before the end of exposure (Table 2).

**Table 2:** Dynamic Light Scattering (DLS) data of Size (nm) and Polydispersity Index (PDI) in exposure medium (Salinity 21 + 0.10 mg/L MWCNTs; Salinity 21 + 1.00 mg/L MWCNTs; Salinity 28 + 0.10 mg/L MWCNTs; Salinity 28 + 1.00 mg/L MWCNTs.) collected in different exposure periods (t0; t7 and t28).

Samples	Size (nm)	PDI
**Salinity 21** + **0**.**10** **mg/L**		
t0_Salinity 21 + 0.10 mg/L	2562.9	1.21
t7_Salinity 21 + 0.10 mg/L	1776.2 (1 I.d.)	0.51
t28_Salinity 21 + 0.10 mg/L	2005.7	0.73
**Salinity 21** + **1**.**00** **mg/L**		
t0_Salinity 21 + 1.00 mg/L	4262.5	1.58
t7_Salinity 21 + 1.00 mg/L	2131.2	0.81
t28_Salinity 21 + 1.00 mg/L	2058.0	0.79
**Salinity 28** + **0**.**10** **mg/L**		
t0_Salinity 28 + 0.10 mg/L	5707.5	1.91
t7_Salinity 28 + 0.10 mg/L	n.d (+3 I.d.)	—
t28_Salinity 28 + 0.10 mg/L	n.d (+3 I.d.)	—
**Salinity 28** + **1**.**00** **mg/L**		
t0_Salinity 28 + 1.00 mg/L	5133.3	1.91
t7_Salinity 28 + 1.00 mg/L	896.0	0.402
t28_Salinity 28 + 1.00 mg/L	2002.5	0.80

I.d.: “Invalid data” (absence of colloidal material into the analyzed sample); n.d.: no data.

Measurements were performed on 1000 µL of suspension in three samples *per* condition, and five analyses *per* sample performed by dynamic light scattering (DLS), using a DelsaTM NanoC Particle Size Analyzer (Beckman Coulter). Each analysis was carried out by performing 120 acquisitions.

#### MWCNTs in sediments

Thermogravimetric analysis (TGA) has been used as innovative method to assess the presence of MWCNT aggregates in the sediments exposed to MWCNT dispersions. To the best of our knowledge no reports describing the use of TGA for such purpose are reported in the literature. TGA may represent an effective method to detect the presence of MWCNTs in the sediments since it records the weight loss of materials upon heating and can distinguish the contribution given by each component of a mixture by applying the derivative operation to the thermogravimetric curve (DTGA analysis). To this aim the degradation behaviour of MWCNTs is unique and might be easily distinguished from that of both inorganic and organic background^66^.

The detection of MWCNTs in sediments has been carried out on 50 mg samples by thermogravimetric analysis (TGA) by using a Mettler Toledo Star-system TGA/SDTA 851e apparatus, with air as the purge gas (60 mL/min) at a scan rate of 10 °C/min. Samples were conditioned for 90 min. at 150 °C prior to analysis in order to remove water.

### Physiological parameters

#### Mortality

Mortality was determined in *H*. *diversicolor* by subtracting the total number of dead individuals at the end of the exposure period (28 days) by the total number of individuals used at the start of the experiment, within each exposure conditions: (control-28 + 0.00 mg/L MWCNTs; control-28 + 0.10 mg/L MWCNTs; control-28 + 1.00 mg/L MWCNTs; 21 + 0.00 mg/L MWCNTs; 21 + 0.10 mg/L MWCNTs; 21 + 1.00 mg/L MWCNTs). Mortality was expressed as percentage.

### Biochemical parameters

The whole body of frozen organisms (3 *per* aquarium, 9 *per* condition) was individually pulverized with liquid nitrogen, divided in 0.2 g aliquots, and used for biochemical analyses. Extractions were performed with specific buffers to determine: (i) energy reserves and metabolic capacity (protein (PROT) content, glycogen (GLY) content, electron transport system (ETS) activity); (ii) activity of antioxidant (superoxide dismutase (SOD); catalase (CAT); glutathione peroxidase (GPx)) and biotransformation (Glutathione S-transferases, GSTs) enzymes; (ii) indicators of cellular damage (lipid peroxidation (LPO) levels, reduced (GSH) and oxidized (GSSG) glutathione content) and iv) neurotoxicity (cholinesterases (ChEs) (Acetylcholinesterase (ATChI-ChE) and Propionylcholinesterase (PTChI-ChE) enzymes activity) markers. Biochemical analyses were performed twice for each sample and parameter (18 measurements *per* condition). The methodologies used to obtain each specific biomarker are described in details in De Marchi *et al*.^67,68^.

### Data analysis

Results of MWCNT characterizations, PROT and GLY contents, ETS activity, SOD, GPx, CAT and GSTs activities, LPO levels, GSH/GSSG, and ChE activities, were submitted to hypothesis testing using permutational multivariate analysis of variance with PERMANOVA + add-on in PRIMER v6. A one-way hierarchical design was followed in this analysis. The pseudo-F *p*-values in the PERMANOVA main tests were evaluated in terms of significance. Significant differences were observed using main test and consequently pairwise comparisons were performed. Values lower than 0.05 were considered as significantly different. The null hypotheses tested were: (i) for each biomarker and for each salinity level, no significant differences existed between MWCNT exposure concentrations or, on the contrary, there is a concentration-dependent toxicity in *H*. *diveriscolor* exposed to MWCNTs material under both salinities; (ii) for each biomarker and for each MWCNTs exposure concentration, no significant differences exist between salinity levels or, on the contrary, salinity shifts altered the toxicity of the MWCNTs material as well as the sensitivity of *H*. *diversicolor* exposed to this contaminant. In figures, different letters represent significant differences among exposure concentrations for each salinity level, with lower case letter for conditions maintained at 28 and upper-case latter for conditions maintained at 21. Significant differences between salinities for each MWCNTs concentration are represented with an asterisk.

A matrix gathering all markers responses *per* exposure concentration was used to calculate a Euclidean distance similarity matrix. This similarity matrix was simplified through the calculation of the distance among centroids matrix based on the exposure concentrations, which was then submitted to ordination analysis, performed by Principal Coordinates (PCO). Pearson correlation vectors of biochemical descriptors (*correlation* > 0.75) were provided as supplementary variables and superimposed on the PCO graph.

## Results

### MWCNTs characterization

#### Water characterization

The water samples obtained after 7 days (d7) and 28 days (d28) of exposure to MWCNTs were almost transparent solutions containing whitish coarse material susceptible to settling and precipitation. In order to assess the dimension of the dispersed material DLS analysis was carried out by using *ad hoc* experimental conditions such as the vigorous and reproducible agitation of each sample before the analysis and the record of a large number of acquisitions during the measurements.

The mean diameter and polydispersity index values, reported in Table 2 were obtained through the cumulant method, which represents the most effective processing tool to reliably compare the DLS data of polydisperse samples and monitor their time evolution. DLS analysis of the samples taken at d7 and d28 did not evidence the presence of particles whose mean dimension was higher than that of the controls indicating the absence of materials deriving from dispersed MWCNTs.

#### Sediment characterization

**Figure 2:**
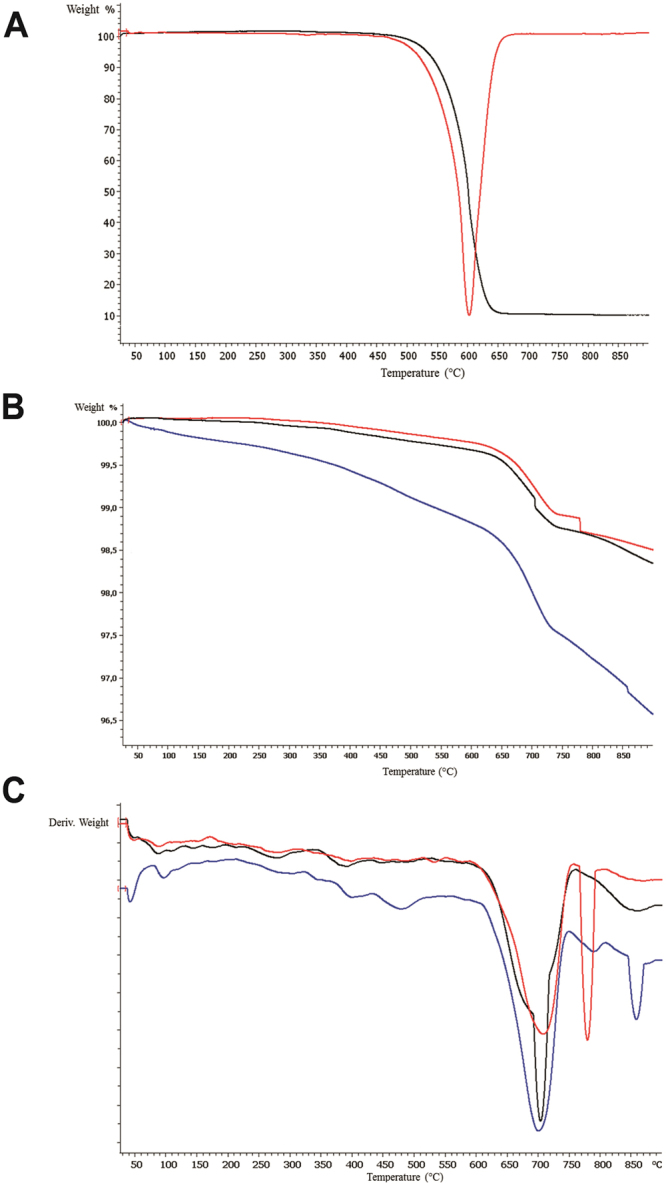
(**A**) Thermogravimetric curve (black line) and derivative of the thermogravimetric curve (red line) of MWCNTs. (**B**) Thermogravimetric curves of sediments: (a) Control Salinity 21 + 0.00 mg/L (black line), (b) t0 Salinity 21 + 1.00 mg/L (red line), (c) t7 Salinity 21 + 1.00 mg/L (blue line). (**C**) DTGA analysis of sediments: (a) Control Salinity 21 + 0.00 mg/L (black line), (b) t0 Salinity 21 + 1.00 mg/L (red line), (c) t7 Salinity 21 + 1.00 mg/L (blue line).

TGA analysis showed the presence of MWCNT aggregates in sediments exposed to MWCNT dispersions stating the feasibility of sediment experimental samples (Fig. 2A).

DTGA analysis evidenced the presence of a negative peak at 600 °C relevant to the maximum rate of degradation of MWCNTs. The thermal characterization was carried out on samples exposed to higher concentration of MWCNT dispersions (1.00 mg/L) and compared to that of the sediment used as control (Fig. 2B).

The weight loss (%) of all the analyzed samples were negligible at 900 °C due to the inorganic nature of the sediments. DTGA analysis of the obtained TGA curves was carried out in order to better understand the steps of degradation (Fig. 2C).

The preliminary DTGA investigation performed on the sediments exposed to MWCNT dispersions did not clearly highlight the presence of negative peaks in correspondence with that of native MWCNTs (600 °C, cf. Fig. 2A). This could be most probably due to the experimental conditions adopted to individuate trace amount of MWCNTs particulate. Further investigation will be devoted to fine tune and optimize the experimental condition in order to detect even traces of MWCNTs in the sediment. However, the use of TGA analysis could represent a promising tool for the detection of MWCNTs in inorganic sediments due to the different degradation behaviour of the CNTs from the background and could represent a valuable technique for their quantification after proper optimization of the method.

### Physiological parameter

#### Mortality

At control (28 + 0.00 mg/L MWCNTs) and 21 + 0.00 mg/L MWCNTs, 100% of survival was recorded after 28 days’ exposure.

Exposures to 0.10 mg/L under salinity 28, mortality was 2.22% while exposures to the same MWCNTs concentration under salinity 21 resulted in 6.66% of mortality.

Exposures to 28 + 1.00 mg/L and 21 + 1.00 mg/L resulted in 11.11% and 7.77% of mortality, respectively.

### Biochemical parameters

#### Energy reserves and metabolic activity

**Figure 3:**
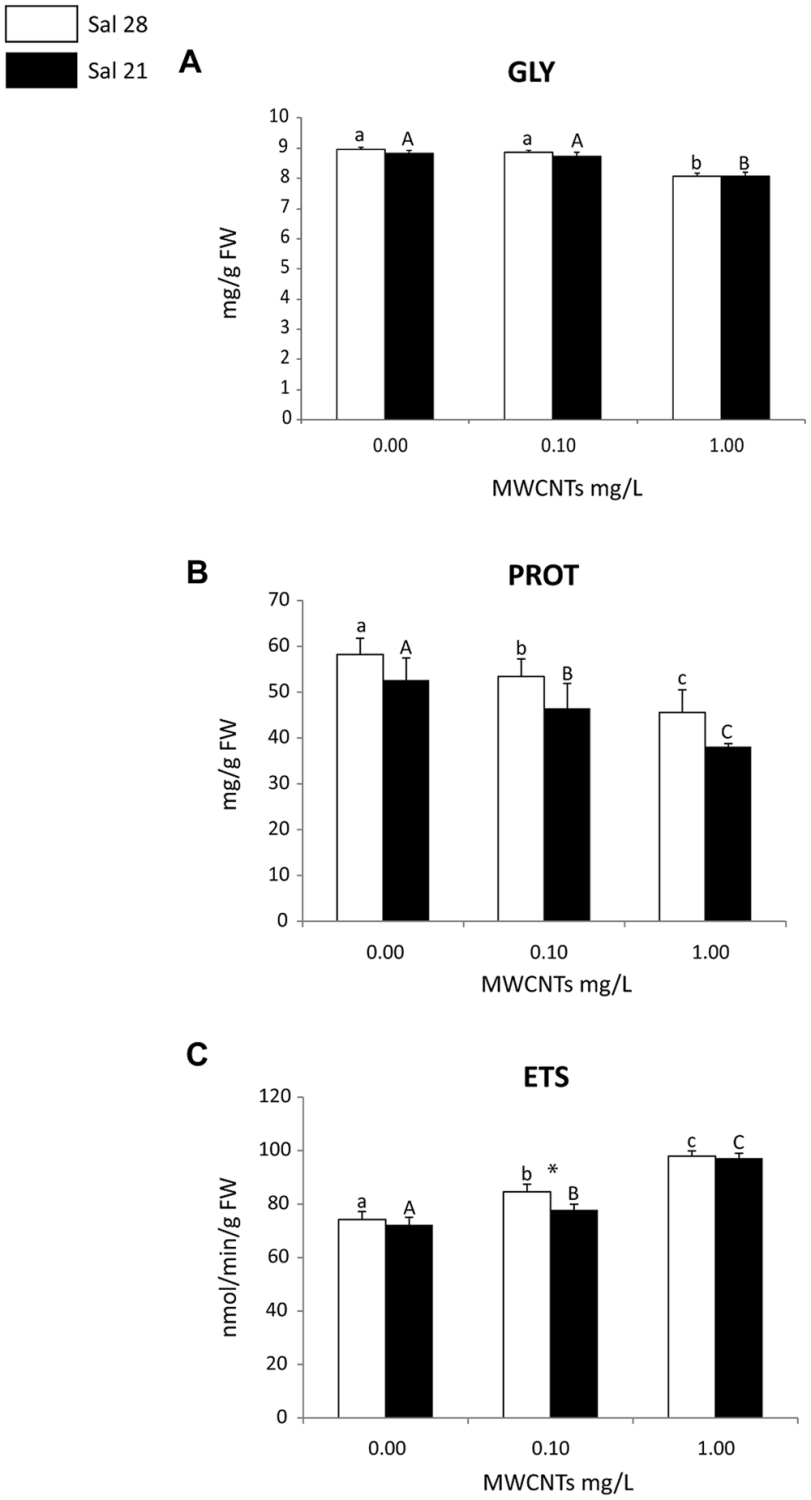
(**A**) Glycogen (GLY) content; (**B**) Protein (PROT) content; (**C**) Electron Transport System (ETS) activity (mean + SD) in *Hediste diversicolor* exposed to different MWCNT concentrations and salinity levels. Significant differences (*p* ≤ 0.05) among MWCNT concentrations are presented with different letters. Significant differences (*p* ≤ 0.05) between salinity levels are presented with asterisks. Sal 28-Salinity 28; Sal 21-Salinity 21.

At both salinities (28 and 21) the lowest GLY content was observed in polycheates exposed to the highest MWCNTs concentration (1.00 mg/L), with significant differences compared to the remaining conditions (control and 0.10 mg/L). No significant differences in GLY content were observed between polychaetes exposed to control and 0.10 mg/L MWCNTs. No significant differences were observed in terms of GLY content when comparing the organisms under both salinities for each of the tested concentrations (control-0.00, 0.10, 1.00 mg/L) (Fig. 3A).

Under both salinities, polychaetes showed significantly lower PROT content when exposed to MWCNTs compared to control individuals, with significant differences among all tested concentrations (Fig. 3B). When comparing organisms exposed to different salinities at each of the tested concentrations (control-0.00, 0.10, 1.00 mg/L), lower PROT content was observed in organisms exposed to the lowest salinity, but no significant differences were observed between salinity levels.

With the increase of MWCNTs exposure concentration, the ETS activity increased in polychaetes under both salinities (control-28 and 21), with significant differences among all exposure concentrations (Fig. 3C). Organisms under the lowest salinity presented significantly lower ETS activity in polychaetes exposed to 0.10 mg/L (Fig. 3C).

#### Antioxidant and biotransformation enzymes activity

**Figure 4:**
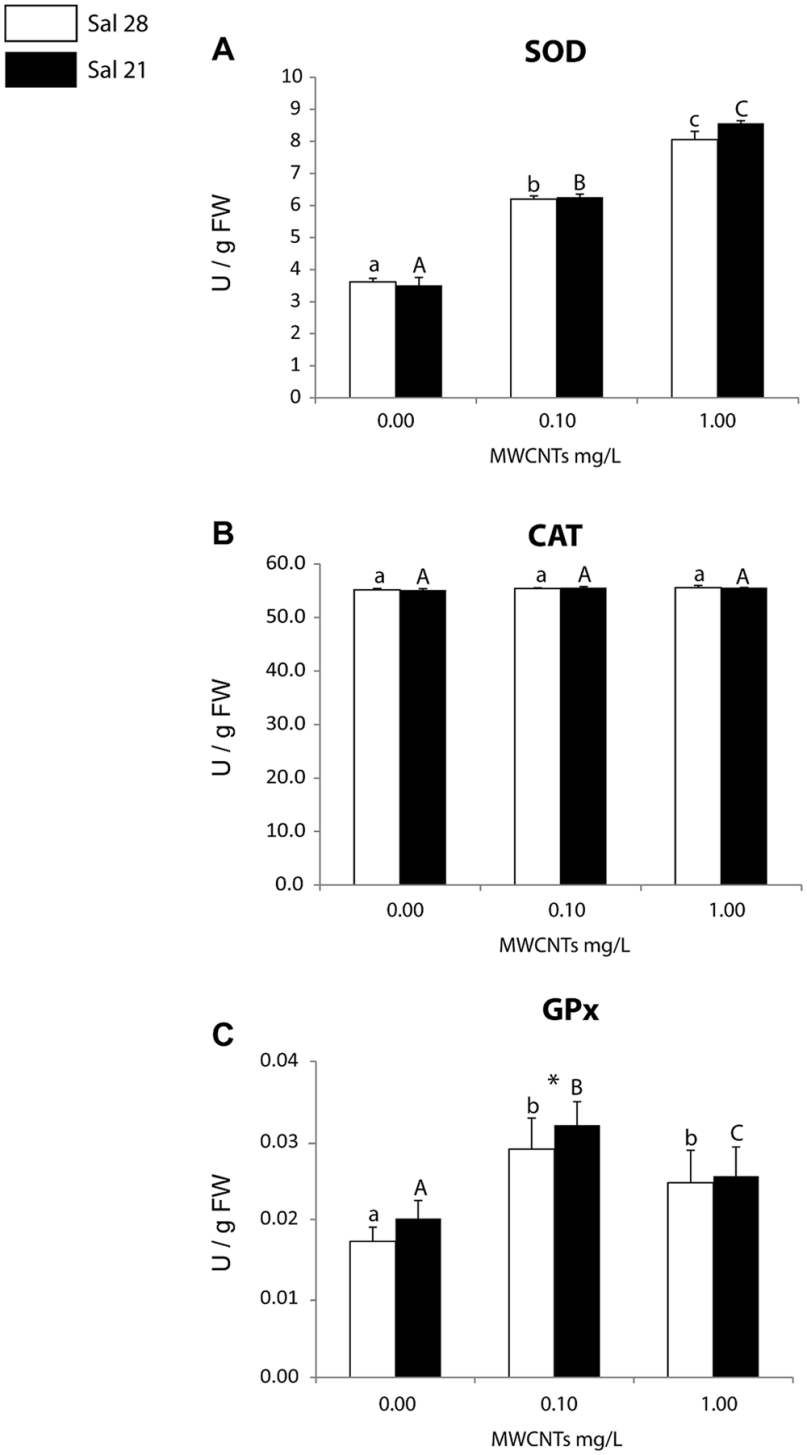
(**A**) Superoxide dismutase (SOD) activity; (**B**) Catalase (CAT) activity; (**C**) Glutathione Peroxidase (GPx) activity (mean + SD) in *Hediste diversicolor* exposed to different MWCNT concentrations and salinity levels. Significant differences (*p* ≤ 0.05) among MWCNT concentrations are presented with different letters. Significant differences (*p* ≤ 0.05) between salinity levels are presented with asterisks. Sal 28-Salinity 28; Sal 21-Salinity 21.

The activity of SOD increased in polychaetes with the increase of exposure concentrations, regardless of the salinity level (control-28 and 21), with significant differences among exposure concentrations (Fig. 4A). Comparing SOD activity in polychaetes at each of the exposure concentrations (control-0.00, 0.10, 1.00 mg/L) no significant differences were observed between individuals exposed to different salinities (28 and 21) (Fig. 4A).

The activity of CAT showed no significant differences among exposure concentrations, both in polychaetes exposed to salinity 28 and salinity 21 (Fig. 4B). Furthermore, at each exposure concentration no significant differences were observed between organisms exposed to different salinities (control-28 and 21) (Fig. 4B).

At both tested salinities, the activity of GPx was significantly higher in polychaetes exposed to MWCNTs (0.10 and 1.00 mg/L) relative to control organisms, with the highest values in polychaetes exposed to 0.10 mg/L (Fig. 3C). In general organisms exposed to salinity 21 presented higher GPx activity, however significant differences between organisms exposed to salinities 21 and 28 were only observed at 0.10 mg/L (Fig. 4C).

**Figure 5:**
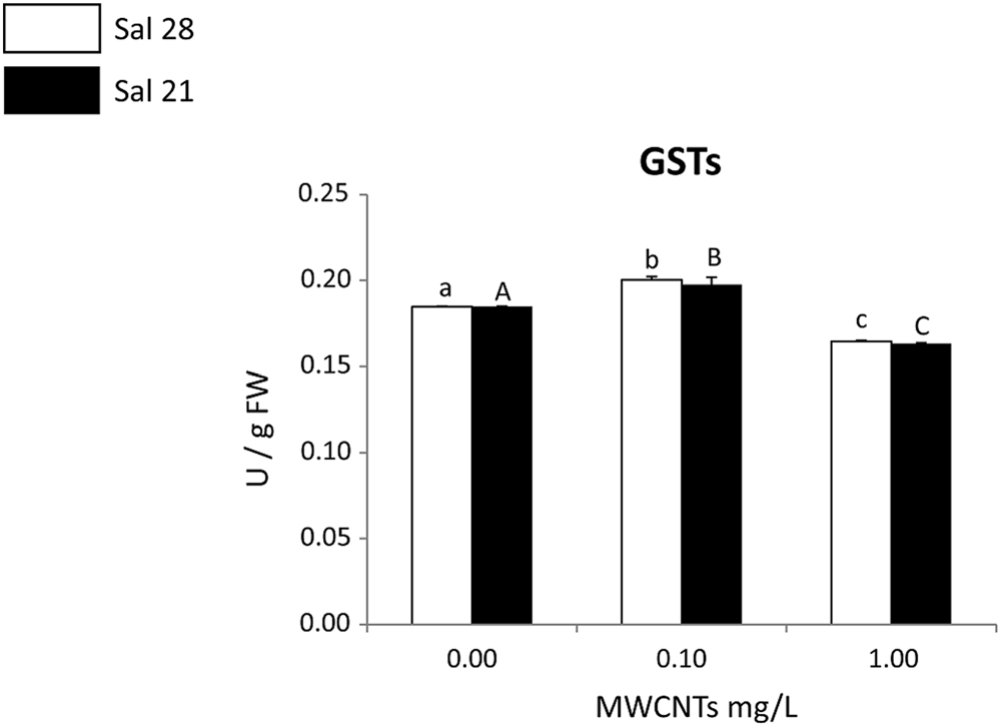
Glutathione S-Transferases (GSTs) activity (mean + SD) in *Hediste diversicolor* exposed to different MWCNT concentrations and salinity levels. Significant differences (*p* ≤ 0.05) among MWCNT concentrations are presented with different letters. Significant differences (*p* ≤ 0.05) between salinity levels are presented with asterisks. Sal 28-Salinity 28; Sal 21-Salinity 21.

Comparing to control, at both salinities 28 and 21, the activity of GSTs significantly increased in polychaetes exposed to 0.10 mg/L while in polychaetes exposed to 1.00 mg/L, the activity of these enzymes significantly decreased (Fig. 5). At each of the exposure concentrations (control-0.00, 0.10, 1.00 mg/L), a similar trend was found, with no significant differences between polychaetes exposed to both salinities (control-28 and 21) (Fig. 5).

#### Indicators of cellular damage

**Figure 6:**
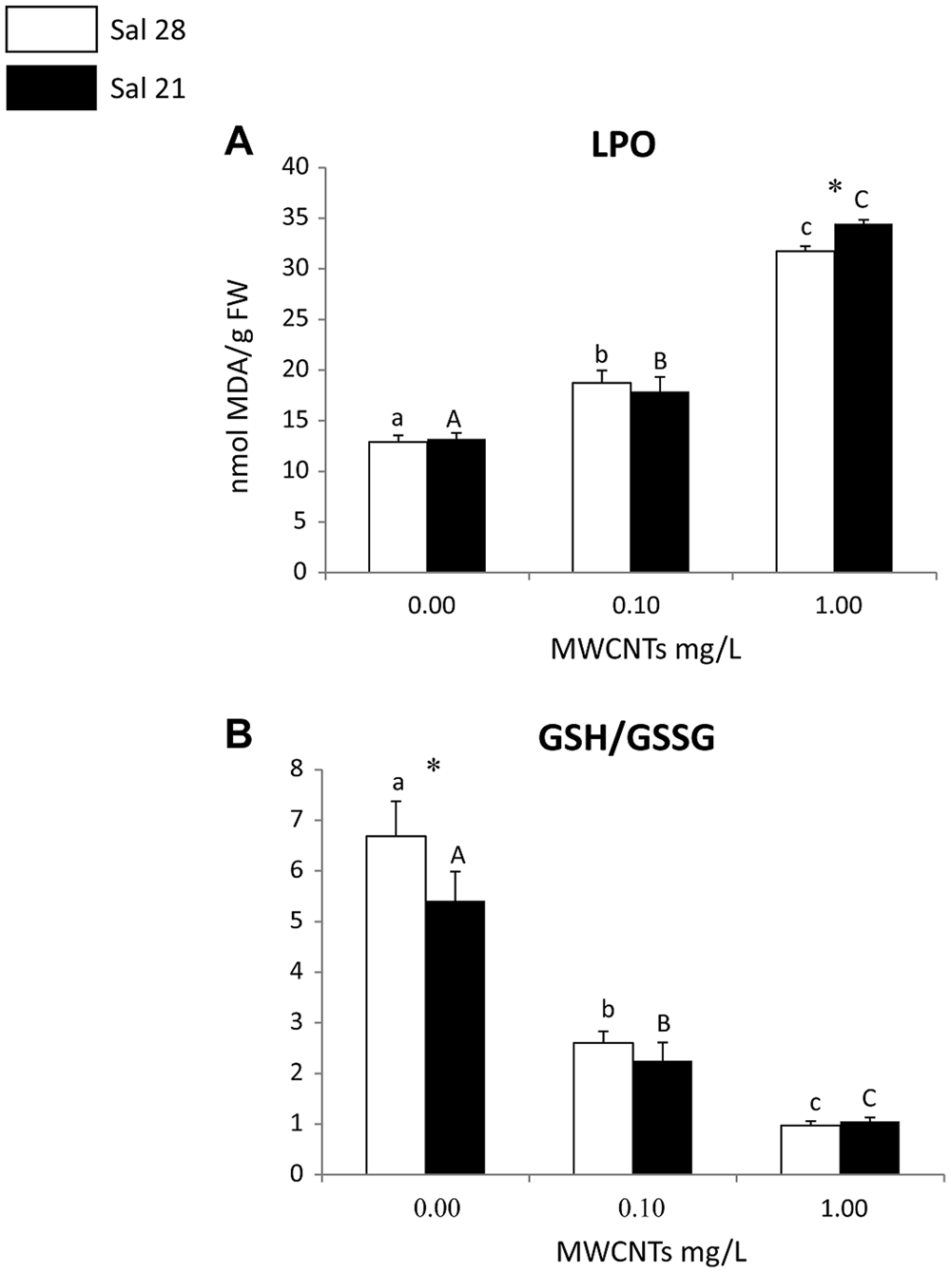
(**A**) Lipid Peroxidation (LPO levels); (**B**) Ratio between reduced and oxidized Glutathione (GSH/GSSG) (mean + SD) in *Hediste diversicolor* exposed to different MWCNT concentrations and salinity levels. Significant differences (*p* ≤ 0.05) among MWCNT concentrations are presented with different letters. Significant differences (*p* ≤ 0.05) between salinity levels are presented with asterisks. Sal 28-Salinity 28; Sal 21-Salinity 21.

Regardless of salinity (control-28 and 21), polychaetes exposed to MWCNT concentrations showed increased LPO levels along the increasing exposure gradient, with significant differences among all conditions (Fig. 6A). Comparing LPO levels in polychaetes under different salinities at each of the exposure concentrations (control-0.00, 0.10, 1.00 mg/L), significant differences were only found at the highest concentration, with organisms under salinity 21 presenting higher LPO values (Fig. 6A).

GSH/GSSG significantly decreased in polychaetes with the increase of exposure concentrations under both salinities, showing significant differences among all concentrations (Fig. 6B). Comparing GSH/GSSG values in organisms at each of the tested concentrations (control-0.00, 0.10, 1.00 mg/L), significantly different values were only observed in organisms at the control conditions, with higher values at salinity 28 (Fig. 6B).

#### Neurotoxicity markers

**Figure 7:**
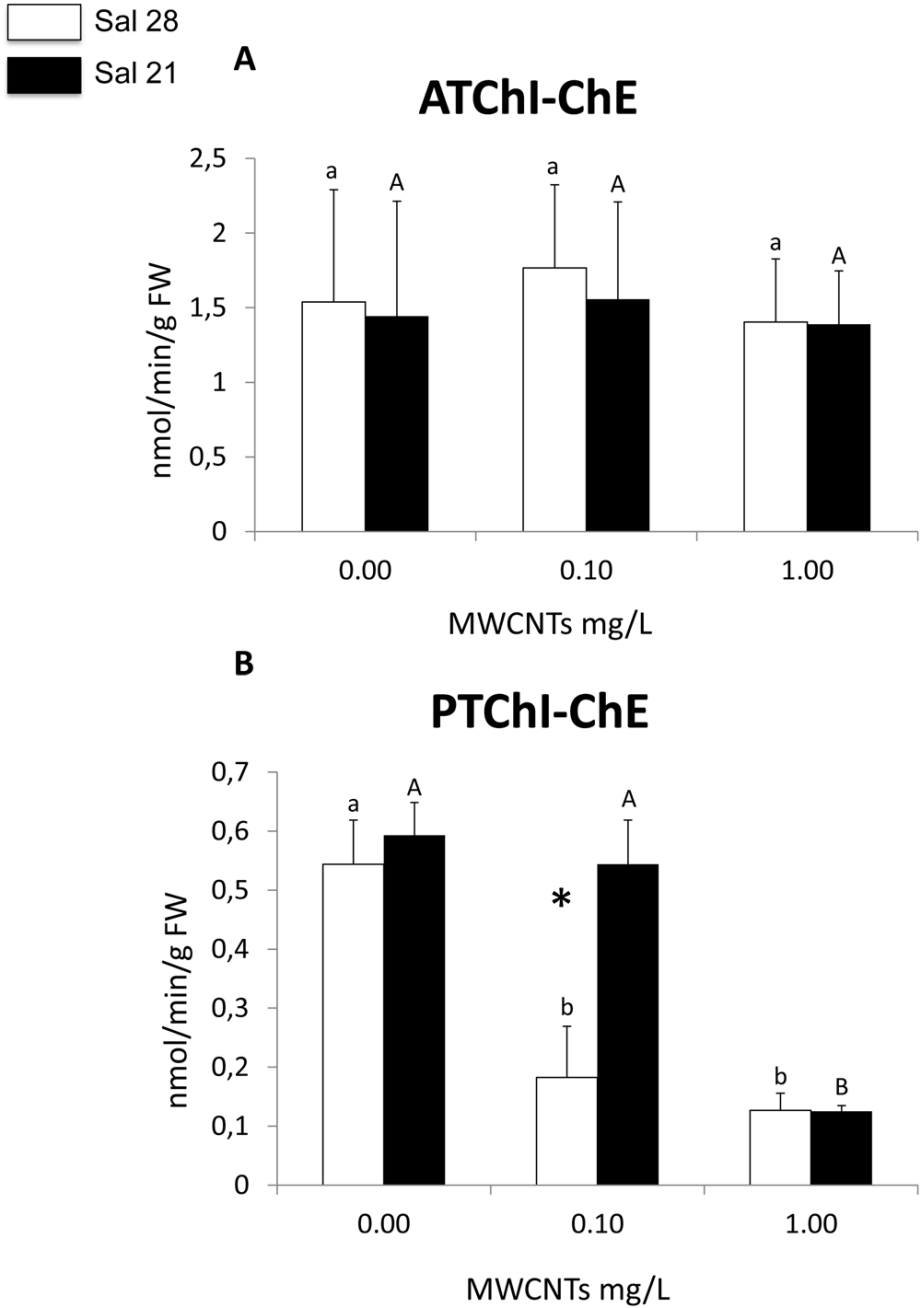
(**A**) Acetylcholinesterase (ATChI-ChE) activity; (**B**) Propionylcholinesterase (PTChI-ChE) activity (mean + SD) in *Hediste diversicolor* exposed to different MWCNT concentrations and salinity levels. Significant differences (*p* ≤ 0.05) among MWCNT concentrations are presented with different letters. Significant differences (*p* ≤ 0.05) between salinity levels are presented with asterisks. Sal 28-Salinity 28; Sal 21; Salinity 21.

No significant differences in ATChI-ChE activity were observed between polychaetes exposed to different concentrations for both salinities tested (Fig. 7A). Moreover, no significant differences were also observed when comparing the ATChI-ChE activity in polychaetes under different salinities at each of the exposure concentrations (Fig. 7A).

Results obtained for PTChI-ChE in organisms exposed to salinity 28 showed significantly lower activity in exposed (0.10 and 1.00 mg/L) compared to non-exposed (control) organisms, while at salinity 21 significantly lower activity was observed only at 1.00 mg/L MWCNTs (Fig. 7B). Significant differences between salinities were only observed in individuals exposed to 0.10 mg/L MWCNTs concentration (Fig. 7B).

### Multivariate analysis

**Figure 8:**
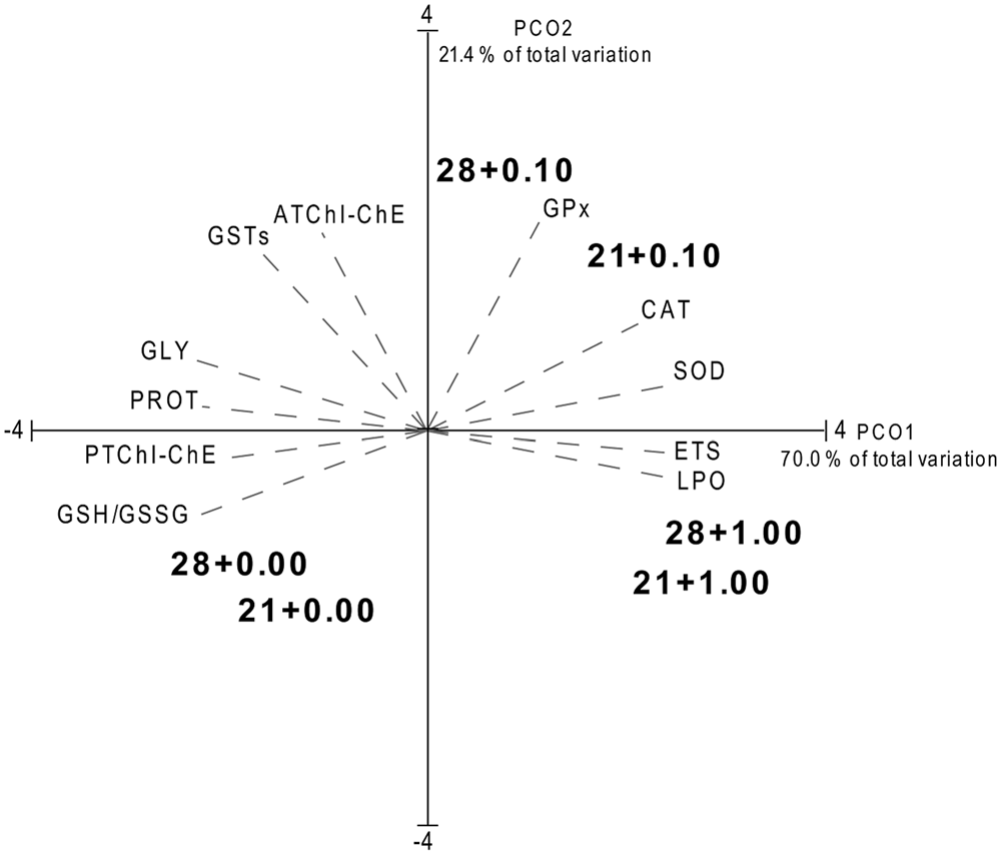
Centroids ordination diagram (PCO) based on biochemical parameters, measured in *Hediste diversicolor* exposed to different MWCNT concentrations and salinity levels. Pearson correlation vectors are superimposed as supplementary variables, namely biochemical data (r > 0.75): Glycogen (GLY); Protein (PROT); Electron Transport System (ETS); Superoxide Dismutase (SOD); Catalase (CAT); Glutathione Peroxidase (GPx); Glutathione S-Transferases (GSTs); Lipid Peroxidation (LPO); Ratio between reduced and oxidized Glutathione (GSH/GSSG); Acetylcholinesterase (ATChI-ChE); Propionylcholinesterase (PTChI-ChE).

Principal coordinates analysis (PCO) graph obtained for *H*. *diversicolor* is shown in Fig. 8 revealing that PCO axis 1 explained 70.0% of the total variation, while PCO axis 2 explained 21.4%. PCO1 separated non-contaminated organisms under control salinity (28) and salinity 21 (28 + 0.00, 21 + 0.00) in the negative side of the axis from individuals exposed to MWCNT concentrations under both salinities (28 + 0.10, 28 + 1.00, 21 + 0.10, 21 + 1.00) in the positive side. PCO2 separates organisms exposed to the lowest MWCNTs concentration (28 + 0.10, 21 + 0.10) under both salinities on the positive side of the axis from the remaining conditions (28 + 0.00, 21 + 0.00, 28 + 1.00, 21 + 1.00) on the negative side. High correlation (r > 0.85) was observed between CAT, SOD, ETS, LPO, GPx and contaminated polychaetes, while ChEs, GSTs, GLY, PROT, GSH/GSSG showed high correlation (r > 0.8) with the uncontaminated individuals exposed to both salinity gradients.

## Discussion

In the present study, *H*. *diversicolor* was exposed to MWCNTs and biochemical alterations induced in the organisms after 28 days of exposure to two salinity levels (28 and 21) at two concentrations (0.10 and 1.00 mg/L) were investigated. Our finding demonstrated that the salinity decrease, may not change the toxicity of MWCNTs neither the sensitivity of organisms to these NMs. Indeed, our results showed that under salinity 21 the toxicity of MWCNTs was similar to the impacts measured under control (28) in terms of oxidative responses, organism’s metabolism and neuro status. Conversely, Kataoka *et al*.^69^ demonstrated salinity-dependent toxicity of NMs in fish. These authors used different concentrations of embryo-rearing medium (ERM) (1x, 5x, 10x, 15x, 20x and 30x) (1 × ERM consisted of 1.0 g NaCl, 0.03 g KCl, 0.04 g CaCl_2_ 2H_2_O and 0.163 g MgSO_4_ 7H_2_O in 1 L of ultrapure water). The results showed that from freshwater (1 × ERM) to seawater (30 × ERM), the toxicity of silver nanocolloids (SNC) to medaka embryos increased. However, the same authors found that at different ERMs, the zeta potentials of a CNTs such as SWCNTs were stable, without differences of particle sizes from freshwater to seawater^54^. In fact, raw CNTs, such as MWCNTs or SWCNTs, are nearly insoluble in any solvent owing to their bundle-shaped configurations^70^ and these properties could partially explain why salinity didn’t increase the toxicity of MWCNTs.

In the present study *H*. *diversicolor* showed increased ETS activity with the increase of exposure concentrations, at both salinity conditions, which might indicate that the increase of metabolic capacity of *H*. *diversicolor* was necessary to activate defence mechanisms to mitigate oxidative stress induced by MWCNTs, leading to the consumption of energy reserves. Our findings are in agreement with the present hypothesis, showing a decrease of energy reserves (measured by GLY and PROT content) when *H*. *diversicolor* specimens were exposed to MWCNTs, a response that was similar under control (28) and low (21) salinity conditions, indicating that the impacts induced by MWCNTs were not altered by salinity shifts. When organisms are exposed to anthropogenic environmental disturbances, they can increase their energy expenditure as a mechanism of cellular protection. Sokolova *et al*.^71^ already demonstrated that marine organisms are able to increase their energy reserves to protect the cell against environmental disturbances. Gagné *et al*.^72^ pointed out that energy expenditure in invertebrates is enhanced when exposed to different pollutants, restricting the amount of energy reserves necessary for survival, homeostasis and reproduction. Also, De Marchi *et al*.^32^ showed that *H*. *diversicolor* and *Diopatra neapolitana* polychaetes presented higher metabolic activity with the increase of NMs exposure concentration, although energetic expenditure was not observed.

The impacts of NMs may also be observed by oxidative stress responses developed by organisms under stressful conditions, which can be assessed by measuring the activity of antioxidant and biotransformation enzymes (such as SOD; CAT; GPx; GSTs), and cellular damage (assessed by LPO) induced due to overproduction of reactive oxygen species (ROS)^24,25^.

In the present study, the activity of SOD, which is the enzyme responsible for the removal of superoxide anion (O_2_^−^) forming hydrogen peroxide (H_2_O_2_)^73^, increased in organisms exposed to MWCNTs, especially at the highest concentration (1.00 mg/L) showing a possible adaptive response to increased ROS production as a consequence of  mitochondrial respiratory chain. On the other hand, CAT, the enzyme that reduces H_2_O_2_ to H_2_O^73^, tended to maintain the activity regardless the MWCNT exposure concentrations under both salinities. Similarly De Marchi *et al*.^32^, using the same carbon NMs, showed that this antioxidant enzyme in *D*. *neapolitana* and *H*. *diversicolor* polychaetes tended to maintain the activity among the exposure conditions. These results may be explained by the fact that the excess of H_2_O_2_ produced by SOD could be eliminated by another antioxidant enzyme with similar capacity (i.e. degradation of H_2_O_2_) and, in the present study, this hypothesis was confirmed by an increase of GPx activity in polychaetes contaminated with 0.10 mg/L MWCNTs compared to non-contaminated. However, in the present study the activity of GPx decreased at the highest exposure concentration (1.00 mg/L) in comparison to the activity observed at 0.10 mg/L. These results could be due to the oxidative stress persisting, or with extreme high ROS levels, which can cause proteins damage and a consequently decrease of the activity of this antioxidant enzyme, as already demostrated by Chatziargyriou *et al*.^74^, where the inhibition of enzyme activity (i.e. GPx) under extreme stressful conditions effects was observed.

Organisms may also present increased activity of GSTs in the presence of contaminantes, a biochemical marker widely used to evaluate the detoxification capacity of organisms catalysing the conjugation of the reduced form of glutathione (GSH) to xenobiotic substrates^73^. Studies, using polychaetes as sentinel species of different quantum dots and metal-based NMs exposures, already showed an increase of these biotransformation enzymes as sensitive biomarkers of defence responses to NMs^24,25,75–79^. However, in the present study the activity of GSTs increased at the lowest tested concentration (0.10 mg/L MWCNTs) but decreased in organisms exposed at the highest concentration (1.00 mg/L MWCNTs), at both salinities. These results may indicate that the activity of this group of enzymes can  be activated up to a certain level of stress (i.e. up to a certain exposure concentration) while  the activity decrease may be associated to a possible inhibition due to the high MWCNTs concentration. This behaviour it is in line with the results of De Marchi *et al*.^80^, which using carboxylated form of MWCNTs showed that the GSTs activity was decreased compared to control at the highest exposure concentration in the clams *Ruditapes philippinarum.* This findings indicated  that this  group of enzymes was not able to  biotransform CNTs into a less toxic excreted substance beyond certain concentration levels, being inhibited at high stress conditions. 

In the present study, although the activation of antioxidant enzymes activities in *H*. *diversicolor* exposed to MWCNTs, the LPO, which is a marker of cellular injury and is an indicator of oxidative damage in cell membranes^73,81^, increased with the increase of exposure concentrations showing that antioxidant mechanisms were not enough to eliminate the excess of ROS as a consequence of the excess of stressful conditions. De Marchi *et al*.^32^ already observed that the activation of antioxidant enzymes activities in *H*. *diversicolor* and *D*. *neapolitana* exposed to MWCNTs was not enough to prevent cellular damage and LPO level increased along the increasing exposure gradient. Also, Anisimova *et al*.^82^, exposed the bivalve *Modiolus modiolus* to 12–14 nm diameter MWCNTs (100 mg/L) for 48 h and demonstrated that CNTs were responsible for the increase of LPO levels and increase of reduced glutathione (GSH), one of the most important antioxidant involved as cellular protector from oxidative stress, acting as a free radical scavenger preventing LPO. Furthermore, our results showed an increase of LPO when organisms were exposed at the highest MWCNTs concentration under low salinity (21). This result may be explained by the fact that higher salinity (in this case 28) decreased the availability of NMs due to their aggregation^54^. Thus, higher availability of NMs to polychaetes exposed to low salinity water media (21) may lead to higher NMs uptake by the organisms causing higher cellular damage (e.g. LPO generation) at this condition.

Stress-induced ROS accumulation is counteracted also by non-enzymatic antioxidant systems that include a variety of scavengers. The role of scavengers is to neutralize ROS by direct reaction with them, and cytosolic reduced glutathione (GSH) is one of the crucial nonprotein thiols that plays an important role in intracellular defence against ROS-induced oxidative damage^83^. GSH is able to neutralize several reactive species through its oxidation, generating oxidized glutathione (GSSG). Thus, the ratio between GSH and GSSG is often used as a marker of oxidative stress^73,84,85^. In the present study, the GSH/GSSG values decreased with the increase of exposure concentrations, at both salinities, indicating that, regardless of different salinity conditions, MWCNTs induced  oxidative stress in *H*. *diversicolor*. Also, in the study performed by De Marchi *et al*.^32^ the concentration of this scavenger, in two polychaete species (*D*. *neapolitana* and *H*. *diversicolor*) exposed to different MWCNT concentrations, strongly decreased among conditions, a pattern that confirms the development of oxidative damage in organisms exposed to CNTs.

Cholinesterase (ChE) activities are a core biomarker of neurotoxicity which interferes with behavioural responses^24^. Physiologically, this class of serine hydrolases are able to remove acetylcholine from the synaptic cleft^86^. Behavioural biomarkers are sensitive tools to assess the impact of contaminants and they are particularly relevant from an ecological point of view^87^. Our results revealed that MWCNTs impaired the hydrolytic activity of ChEs. In fact, a significant inhibition of PTChI-ChE activity in *H*. *diversicolor* under MWCNTs exposure compared to control condition was proved. Previous studies have already used these neurotoxicity markers as a useful tool to evaluate the impact of different metal-based nanomaterials on polychaetes (Ag NPs, Au NPs, CuO NPs, CdS NPs, ZnO NMs)^25,77,88^ and recently also carbon nanomaterials (MWCNTs NMs)^32,89^ showing, in most of the cases, a inhibition of ChEs activities in polychaetes exposed to NMs demonstrating a possible neurotoxicity induction when organisms are exposed to these emerging pollutants.

Given the ecological relevance of polychaetes, any significant injury on these key organisms may bring consequent impacts at the ecosystem level. Thus, our findings provide valuable information regarding the potential risk of CNTs  to *H*. *diversicolor* considering the projected increase of nanotechnology and industrial uses of NMs and consequent release to aquatic ecosystems.
